# Identification and Genetic Analysis of Species D Rotaviruses in Pangolin Samples

**DOI:** 10.1155/tbed/1773821

**Published:** 2024-11-26

**Authors:** Kai Wang, Shasha Liu, Xiaotong Liang, Wanke Hu, Zhenyu Wen, Jiayi Wang, Xianghe Wang, Fuyu An, Ziqiao Chen, Haikuo Yan, Hongmei Yan, Lei Wang, Xiaoai Zhang, Jieshi Yu, Wen-Kang Wei, Yan Hua

**Affiliations:** ^1^Guangdong Provincial Key Laboratory of Silviculture, Protection and Utilization, Guangdong Academy of Forestry, Guangzhou, Guangdong, China; ^2^State Key Laboratory of Swine and Poultry Breeding Industry, Agro-biological Gene Research Center, Guangdong Academy of Agricultural Sciences, Guangzhou, Guangdong, China; ^3^College of Veterinary Medicine, South China Agricultural University, Guangzhou, Guangdong, China; ^4^College of Coastal Agricultural Sciences, Guangdong Ocean University, Zhanjiang, Guangdong, China

**Keywords:** metagenomic sequencing, pangolin, phylogenetic analysis, rotavirus D, virome

## Abstract

Pangolins have been found to carry severe acute respiratory syndrome coronavirus 2 (SARS-CoV-2)-related coronaviruses. In light of this discovery, interest has been piqued in viromes of these heavily trafficked wild animals. In this study, we performed viral metagenomic sequencing to explore viromes of both confiscated dead pangolins and captive healthy pangolins. Sequence reads of vertebrate-associated viruses in *Herpesviridae*, *Retroviridae*, *Iridoviridae*, *Reoviridae*, *Arenaviridae*, and *Flaviviridae* were detected in confiscated dead pangolins. A novel rotavirus (RV) (*Reoviridae*), showing a high degree of genetic similarity to the RV species D (RVD) that was previously unreported in mammals, was further confirmed by using reverse transcription-polymerase chain reaction (RT-PCR) and Sanger sequencing. Three out of 18 samples from the confiscated dead pangolins were positive for genomic sequences of the novel RV. Importantly, sequence alignments and phylogenetic analyses demonstrated that these RV strains genetically belonged to the RVD. Nevertheless, these novel RVD strains were divergent from known RVD strains that have been found only in Avian. They formed a separate genetic cluster. Five serial passages were attempted to isolate the RV, but no live virus was obtained. In addition, fecal samples were collected from healthy pangolins (*n* = 41) in our institution and screened for RVs by viral metagenomic sequencing and RT-PCR. In these fecal samples, neither species D nor previously identified species A RVs were detected. This study reported RVDs in pangolin samples for the first time to our knowledge. Identifiability disagreements between wild and captive pangolins highlight the need for further exploration into pangolin viruses to better understand their emergence and transmission potential.

## 1. Introduction

Pangolins are now believed to be the most heavily poached and trafficked mammal in the world. In recent years, they have attracted increasing globe attention because of their potential role as hosts for coronaviruses posing a threat to public health [1–4]. Virome studies have revealed that wild pangolins can carry many vertebrate—and arthropod-associated viruses, including those in *Coronaviridae*, *Picornaviridae*, *Parvoviridae*, *Flaviviridae*, *Herpesviridae*, *Paramyxoviridae*, *Reoviridae*, *Retroviridae*, *Circoviridae*, *Polycipiviridae*, *Polydnaviridae*, *Baculoviridae*, *Dicistroviridae*, *Adintoviridae*, *Lispiviridae*, *Artoviridae*, *Astroviridae*, *Rhabdoviridae*, *Orthomyxoviridae*, *Phasmaviridae*, and *Phenuiviridae* [5–9]. It is suggested from these results that pangolins may be considered susceptible hosts and may play a role in the emergence or re-emergence of diverse viruses. However, metagenomics does not provide direct evidence that pangolins can be infected with certain viruses. To date, a number of live viruses have been isolated from pangolins, including the severe acute respiratory syndrome coronavirus 2 (SARS-CoV-2)-related coronavirus [3], the bat Middle East respiratory syndrome (MERS)-like coronavirus [2], the canine parvovirus [10, 11], and the parainfluenza virus 5 (PIV5) [12].

Rotaviruses (RVs) belong to the *Reoviridae* family and are widely prevalent worldwide. Twelve different species of RVs (A-L) are currently classified according to sequences of viral capsid protein VP6 [13–16], and nine RV species (except for RVE, RVK, and RVL) have been officially included by the International Committee on Taxonomy of Viruses (ICTV) (Virus Taxonomy: 2022 Release, https://ictv.global/taxonomy). RVA–RVC, as well as RVH, infects both humans and multiple animals [17–21]. So far, the other RV species have only been found from animals [13–15, 22, 23]. RV species D (RVD) and RVF–RVG have been identified in avian [22, 23]; RVE has been detected in swine, but it has been removed from the official ICTV taxonomy due to the lack of sequence data or an isolate of this species (https://ictv.global/taxonomy). RVI has been found in felines and canines [15]; RVJ has been found in bats [14]; RVK and RVL have been found in shrews [13]. RVs are nonenveloped, double-stranded RNA viruses consisting of 11 genomic segments [24]. The total genome size is 18–19 kb, and individual genomic segments range from 667 to 3305 bp in length [25, 26]. Each of these genomic segments encodes one of proteins that are viral structural proteins VP1–VP4 and VP6–VP7, and viral nonstructural proteins NSP1–NSP5. In some RV strains, the NSP5-encoding segment produces an additional nonstructural protein NSP6 [27]. Generally, VP4, VP6, and VP7 determine RV antigenicity, where VP6 determines RV serogroups (species) [16], and VP4 and VP7 determine RV serotypes [28].

RVs are important pathogens responsible for diarrheal disease in humans and animals. In a virome study, the human-associated RVA was detected in pooled tissue samples of smuggled dead pangolins [7]. The assembled sequences of individual segments of these pangolin RVA strains showed 52.0%–87.7% nucleotide (NT) similarity with those of known RVA strains, which suggested that these pangolin RVA strains might be classified as new genotypes [7]. In another virome study, sequence reads mapping to RVs were identified in samples from confiscated healthy pangolins [8]. It appears from these studies that pangolins may be susceptible to RVs, and the spread of RVs may occur in pangolin populations. To identify the genetic diversity and possible prevalence of RVs in pangolins, we conducted a small-scale epidemiological study of RVs in confiscated dead pangolins as well as captive healthy pangolins. It will contribute to the study of pangolin infectious disease and rescue operations in the future.

## 2. Materials and Methods

### 2.1. Sample Collection

From 2018 to 2023, our institution received around 200 confiscated dead pangolins. Most of them were not properly stored. Samples were eventually collected from 18 well-preserved dead pangolins. Available tissues, including turbinate, larynx, trachea, lung, and/or intestine from the same animal, were pooled and thoroughly homogenized in Dulbecco's Modified Eagle Medium (DMEM) and designated as one single sample. Nowadays, 41 rescued pangolins, including both Malayan pangolins (*Manis javanica*) (*n* = 20) and Chinese pangolins (*Manis pentadactyla*) (*n* = 21), were kept in our facility. They all looked healthy, and we took 49 fresh fecal samples from them. All the samples were taken by veterinarians or under the supervision of veterinarians.

### 2.2. Viral Metagenomics

The supernatants were obtained from pangolin tissue homogenates or fecal solutions after centrifuge (2000 rpm, 10 min). Total RNAs were extracted from the supernatants of individual pangolin tissue samples or fecal samples using the TRIzol LS reagent (Invitrogen). For virome in confiscated dead pangolin samples, six RNA samples of the highest quality were mixed using 300 ng from each sample. For virome in fecal samples from captive healthy pangolins, six RNA samples for the Malayan or Chinese pangolins were randomly selected and pooled using 300 ng from each sample. The sequencing libraries were constructed using the ALFA-SEQ OnePot Pro DNA Library Prep Kit (Illumina) (FINDROP) and were then sequenced on the Illumina platform (NovaSeq 6000) using paired 150 bp reads. After the original data were unloaded, a series of processing of the data were conducted through Trimomatic software to obtain high-quality clean reads. Host-derived and ribosomal RNA (rRNA)-matched sequence reads were removed by performing Burrows–Wheeler Alignment (BWA) software (v0.7.17). Sequence reads of viruses were gained by mapping the clean reads to the viral reference sequences (RefSeqs) in the GenBank nonredundant NT database via the BWA software. The resulting sequence reads were then de novo assembled into contigs by employing the Megahit programme (v1.2.9). The contigs longer than 300 bp were subsequently compared to the viral RefSeq derived from National Center for Biotechnology Information (NCBI) nonredundant NT and protein databases by BLASTn (v2.9.0+) and BLASTx (v2.9.0+), respectively, with a cutoff *E*-value 1 × 10^−5^. To gain viral sequences, NCBI Taxonomy information was acquired for the top 50 blast hits of each contig. If more than 20% of the top 50 blast hits were nonviral sequences (annotated as Eukaryota, Bacteria, or Archaea), the contig was considered as the nonviral sequence; otherwise, the contig was identified as the potential virus-associated sequence.

### 2.3. PCR Detection and Sanger Sequencing

The isolated RNA was reverse transcribed into complementary DNA (cDNA) by using the HiScript III 1st Strand cDNA Synthesis Kit (Vazyme). Briefly, the RNA was placed in a microcentrifuge tube (0.2 mL) and incubated at 65°C for 5 min. Since the RNA was possibly contaminated with trace amounts of genomic DNA (gDNA), DNase I was added to the reaction to eliminate gDNA. After the removal of gDNA, a 20 µL of reverse transcription reaction with the random hexamer was prepared and proceeded following the manufacturer's thermal cycling protocols. For RV detection, reverse transcription-polymerase chain reaction (RT-PCR) assays with primers (Table 1) targeting the VP6 gene were performed. For amplification of RV genomic segments, a series of conserved RV-specific primers (Table 1) were designed and synthesized. PCR was performed by using Taq Master Mix Kit (Vazyme) following manufacturer's standard protocols. RT-PCR products were purified (Gel DNA Extraction Kit, Omega) and sent to Sanger sequencing using the RV-specific primers. Sequences of individual gene segments were proofread and assembled manually and then deposited into GenBank (PP319611-PP319630).

### 2.4. Sequence Alignment Analysis

Sequence alignments between new and known RVs were performed by conducting the BLAST tool (http://ncbi.nlm.nih.gov). In terms of individual gene segments, the known RVs showing the highest NT similarity to the new RVs were listed in Table 2.

### 2.5. Phylogenetic Analysis

Gene sequences of RVs were retrieved from the GenBank database (accessed on January 25, 2024). The sequences obtained in this study were aligned with the downloaded sequences by ClustalW in MEGA-X [29]. Gene sequences less than 600 bp were excluded from the analysis. Phylogenetic trees were created by using the maximum likelihood method with 1000 bootstrap replicates in MEGA-X [29]. The pairwise patristic distances between sequences were exported following the phylogenetic analysis. The average pairwise patristic distances between VP6 gene sequences within or among individual RVD clusters were further calculated and analyzed in Microsoft Excel.

## 3. Results

### 3.1. Identification of Viruses in Pangolin Samples

#### 3.1.1. Virome of Confiscated Dead Pangolin Samples

**Figure 1:**
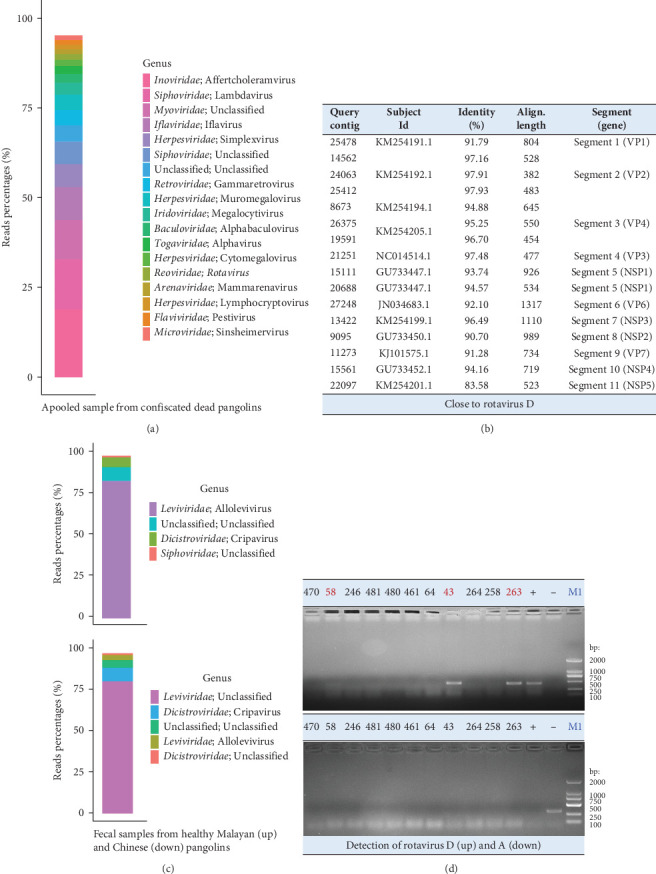
Virus identification in pangolin samples. The profile of viral sequence reads in confiscated dead pangolin samples (a) or fecal samples of healthy Malayan (c, up) and Chinese (c, down) pangolins. Each colored box in the panel represents the percentage of reads belonging to the given virus genus based on the BLASTn analysis. The virus genus with sequence reads greater than 1% was shown. Reads percentage = (sequence reads of a virus genus/total viral sequence reads) ×100. The identified virus genus RV (*Reoviridae*) was highlighted with the bolded font. (b) Contigs mapping to the RVD. Contigs longer than 300bp were compared to the viral reference sequences by BLASTn and BLASTx, respectively, with *E*-value less than 1 × 10^−5^. Contigs match to RVs were listed. Information on the subject sequence ID, identity and aligned length between the query contig and subject sequence, and gene segment were also displayed. (d) Agarose gel electrophoresis showing the products resulting from RT-PCR on dead pangolin samples using the primers targeting the VP7 gene of RVD (up) or the VP6 gene of RVA (down). RV-positive samples were labeled and underscored with the red color. “–” represents the negative control and “+” represents the positive control. RT-PCR, reverse transcription-polymerase chain reaction; RV, rotavirus; RVD, rotavirus species D.

High-quality nucleic acids extracted from confiscated dead pangolin samples were pooled for viral metagenomic sequencing. A total of 17 million high-quality reads were obtained. After removing host-derived reads and subtracting rRNA-matched reads, 4.94 million reads (29%) remained. These sequence reads were then compared to the virus RefSeq database (NT sequence database with entries from all classical divisions of GenBank, European Molecular Biology Laboratory [EMBL], and DNA Data Bank of Japan [DDBJ]). Eventually, a total of 26,374 viral sequence reads were identified, representing 0.15% of the total reads. Since bacteriophages are common in metagenomic sequencing, sequence reads (51.15%) mapping to viruses in *Inoviridae*, *Siphoviridae*, *Myoviridae*, and *Microviridae* were not further analyzed (Figure 1a). We identified 14.02% of the viral sequence reads belonging to insect-associated viruses, including those in *Iflaviridae*, *Baculoviridae*, and *Togaviridae*; and 25.37% of the viral sequence reads belonging to vertebrate-associated viruses, including those in *Herpesviridae*, *Retroviridae*, *Iridoviridae*, *Reoviridae*, *Arenaviridae*, and *Flaviviridae* (Figure 1a). The remaining 9.46% of the viral sequence reads was unclassified or unaccounted. Following *de novo* assembly, a novel RV with the genome coverage higher than 50%, sharing a high degree of genetic similarity with the RVD, was further investigated (Figure 1b).

#### 3.1.2. Virome of Fecal Samples From Captive Healthy Pangolins

RVs are important etiologic agents of gastrointestinal diseases in animals worldwide. Besides this study, RVs have been found in both healthy and dead or diseased pangolins by the next-generation sequencing [7, 8]. To survey if RVs, including these newly identified RVs and previously reported species A RVs, are present in captive live pangolins in our institution, we conducted viral metagenomic sequencing for the pangolin fecal samples. After quality control and removal of host-derived reads as well as rRNA-matched reads, 1,475,693 (8.27% of the total reads) and 1,825,400 (9.38% of the total reads) viral sequence reads were generated from fecal samples of healthy Malayan and Chinese pangolins, respectively. However, 90% or more of the viral sequence reads belonged to bacteriophages (in *Leviviridae* and *Siphoviridae*) and insect-associated viruses (in *Dicistroviridae*) (Figure 1c). Less than 1% of the viral sequence reads mapped to vertebrate-associated viruses (data not shown).

#### 3.1.3. Virological Surveillance for RVs in Pangolin Samples by RT-PCR

The tissue samples (*n* = 18) from confiscated dead pangolins and fecal samples (*n* = 49) from captive healthy pangolins were examined for species A and D RVs by RT-PCR assay. Three tissue samples (Nos. 43, 58, and 263) were identified positive by amplification of the RVD VP7 gene fragment (Figures 1d and *Supporting Information 1*: Figure S1). Each positive sample was a mixture of trachea, lung, and intestine tissues from the same animal. None of tissue samples were tested positive by amplification of the species A RV VP6 gene fragment (Figures 1d and S2b). Furthermore, neither species D nor species A RVs were detected in these fecal samples (*Supporting Information 1:* Figure S1). These results suggest that RVs are not currently prevalent in captive live pangolins at our institution, even though they have been found in confiscated dead pangolin samples by this and other studies.

### 3.2. Amplification, Genome Sequencing, and Genetic Characterization of RVs in Pangolin Samples

#### 3.2.1. Amplification, Genome Sequencing, and Alignment Analysis

Individual genomic segments were amplified from the RV-positive samples. The purified RT-PCR products were Sanger sequenced. The obtained sequences were proofread and assembled. Partial sequences of gene segments VP1, VP2, VP4, VP6–VP7, and NSP1–NSP3 were acquired from the sample 263 (*Supporting Information 2*: Figure S2), partial sequences of gene segments VP1, VP2, VP4, VP6–VP7, and NSP1 were obtained from the sample 43 (*Supporting Information 2*: Figure S2), and partial sequences of gene segments VP1, VP2, VP4, VP6, NSP1, and NSP3 were obtained from the sample 58 (*Supporting Information 2*: Figure S2). All these sequences were submitted to GenBank (designated as RVD/Pangolin/China/43/2023, RVD/Pangolin/China/58/2023, and RVD/Pangolin/China/263/2023, respectively; accession number: PP319611-PP319630).

The alignment results indicated that sequences of these RVs shared the highest NT similarities to those of chicken RVDs (Table 2). The sequence similarities of individual genomic segments between RVs found in this study and the most homologous RVs to them ranged from 89.01% to 97.59% (Table 2). Among these genomic segments, the VP2 gene segments of RVD/Pangolin/China/43/2023, RVD/Pangolin/China/58/2023, and RVD/Pangolin/China/263/2023 displayed the highest sequence similarities (96.39%–97.59%) to that of RVD/Chicken/South Korea/D62/2013 (KM254192), while VP7 segments of RVD/Pangolin/China/43/2023 and RVD/Pangolin/China/263/2023 displayed the lowest sequence similarities (89.01% and 89.97%) to that of the RVD/Chicken/Brazil/BRA/98/2010 (KJ101578) and RVD/Chicken/Brazil/BRA/85/2010 (KJ101575), respectively (Table 2). These data suggest that the pangolin RVs are close to species D chicken RVs but are genetically distinct from them.

#### 3.2.2. Phylogenetic Analysis

**Figure 2:**
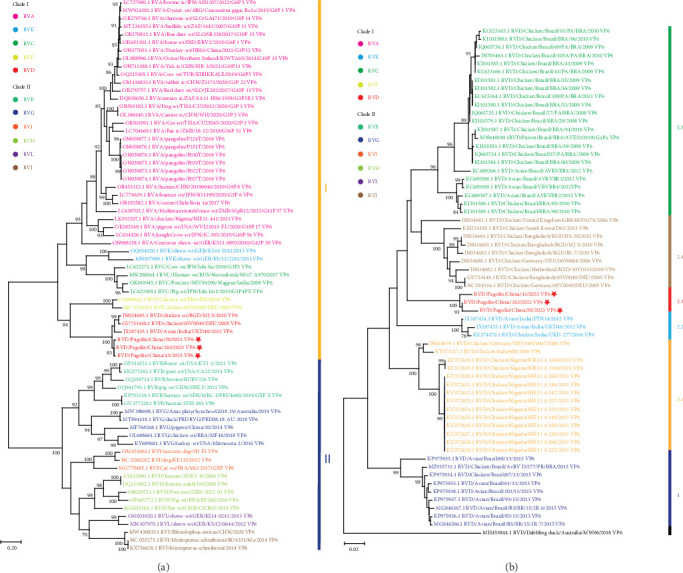
Phylogenetic analyses based on the RV VP6 gene sequences. (a) Maximum-likelihood phylogenetic tree of RVs from different species. RVs were divided into two major clades: I (marked by a vertical yellow line) and II (marked by a vertical blue line). RV species A, C, D, F, and K in clade I, and B, G, H, I, J, and L in clade II were labeled with different colors. (b) Maximum-likelihood phylogenetic tree of RVDs. RVD clusters were marked with different colors. The RV VP6 gene sequences were aligned by ClustalW in MEGA-X. Phylogenetic trees were constructed by maximum-likelihood method with 1000 bootstrap replicates. Bootstrap scores less than 80 were not shown. Scale bar signifies the number of substitutions per site. Individual phylogenetic clusters were noted by different colors. RVs identified from pangolin samples were highlighted with bolded font, and the RV strain reported by this study was marked with a star. RV, rotavirus; RVD, rotavirus species D.

In terms of the VP6 gene sequences, RVs were divided into two major clades (I and II) (Figure 2a). The clade I included RV species A, C, D, F, and K, while the clade II consisted of RV species B, G, H, I, J, and L (Figure 2a). RVs identified in this study clustered together within the RVD, which were phylogenetically distant from previously reported pangolin species A RVs (Figure 2a). These results confirmed that the newly identified RVs in pangolin samples were RVDs.

To further understand genetic relationships among RVDs, the obtained gene segments of RVD in pangolin samples were individually analyzed through phylogenetic analysis together with RVD gene segments retrieved from the GenBank database (accessed on January 25, 2024). According to VP6 gene sequences, RVD strains could be classified into six clusters (1 and 2.1–2.5) and one distinct strain (Figure 2b). The mean pairwise patristic distances between sequences within individual clusters were lower than 0.05, which were 0.02, 0.012, 0.011, 0.02, 0.041, and 0.029, respectively (*Supporting Information 3*: Figure S3). In contrast, the average pairwise patristic distances between sequences among clusters and the distinct strain were higher than 0.1, ranged from 0.114 to 0.267 (*Supporting Information 3*: Figure S3). The distinct strain exhibited the greatest pairwise patristic distances (0.224–0.267) to strains in all clusters, and strains in cluster 1 showed the secondly greatest pairwise patristic distances (0.138–0.168) to strains in the other clusters (*Supporting Information 3*: Figure S3). Under these criteria, the RVD strains identified in this study were classified into the separate cluster 2.3, with the pairwise patristic distances 0.12–0.167 to strains in the other clusters (*Supporting Information 3*: Figure S3).

**Figure 3:**
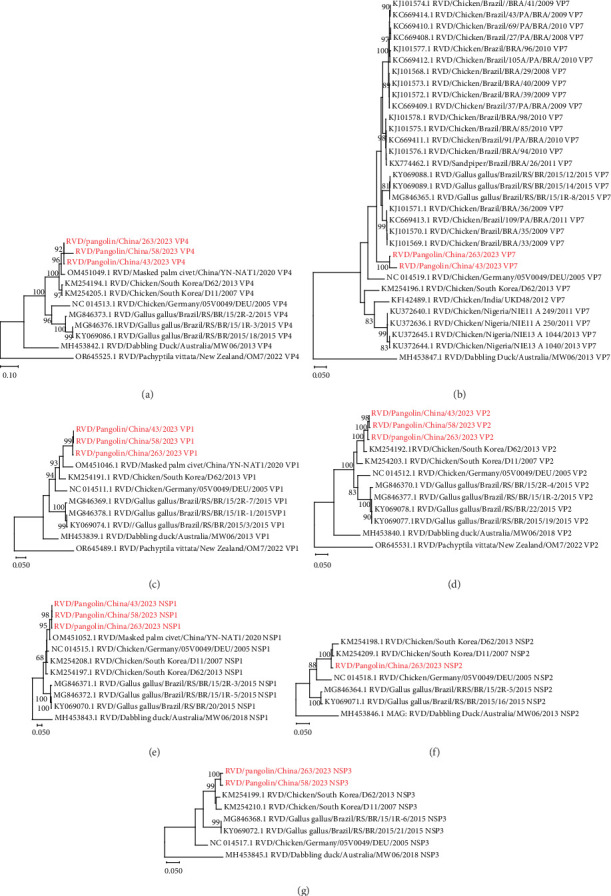
Phylogenetic analyses based on the RV VP1, VP2, VP4, VP7, and NSP1–NSP3 gene sequences. (a–g) Maximum-likelihood phylogenetic trees of RVDs. The individual gene sequences of RVDs were aligned by ClustalW and phylogenetically analyzed by maximum-likelihood method in MEGA-X, with 1000 bootstrap replicates. Bootstrap scores of at least 80 were shown. Scale bar represents the number of substitutions per site. The RV strain reported by this study was marked with red color. RV, rotavirus; RVD, rotavirus species D.

Phylogenetic trees were also generated for other RVD gene segments (VP1, VP2, VP4, VP7, and NSP1–NSP3) (Figure 3). In each phylogenetic tree, except for the distinct strain (RVD/Dabbling duck/Australia/MW06/2018 or RVD/*Pachyptila vittata*/New Zealand/OM7/2022), all RVD strains could be divided into two major clusters (Figure 3). One was represented by the strain RVD/Chicken/South Korea/D62/2013, and the other one was mainly composed of Brazilian chicken RVD strains (Figure 3). In VP1, VP2, VP4, NSP1, NSP2, and NSP3 gene-derived phylogenetic trees, RVD strains identified in pangolin samples were phylogenetically existed in the RVD/Chicken/South Korea/D62/2013-represented cluster (Figure 3a,c–g). However, in the VP7 gene-derived phylogenetic tree, RVD strains from pangolin samples were phylogenetically closer to the Brazilian chicken RVD strains than to the RVD/Chicken/South Korea/D62/2013 (Figure 3b).

## 4. Discussion

Virome studies have identified various viruses in samples from wild pangolins (dead, diseased, or healthy) [7–9]. Successful isolation of live viruses together with serological evidences has demonstrated that pangolins can be naturally infected with the SARS-CoV-2-related coronavirus, the bat MERS-like coronavirus, the canine parvovirus, and the PIV5 [2, 3, 10–12]. The infection of pangolins with other viruses has not yet been confirmed. Further research is needed to determine whether exposure to diverse viruses can lead to infection, transmission, and even disease in pangolins. Our institution is the wildlife rescue and conservation facility permitted to care and breed pangolins. Figuring out the infectivity and prevalence of viruses in pangolins is necessary for the prevention and control of pangolins' infectious diseases.

In this study, with the focus on RVs, we conducted the surveillance study for viruses in confiscated dead pangolins as well as conserved healthy pangolins. Although RVs were identified in dead pangolin samples by the metagenomic sequencing and RT-PCR (Figures 1a,d and *Supporting Information 2*: Figure S2), no prevalence of RVs was observed in fecal samples from our conserved healthy pangolins (Figures 1d and S2). Furthermore, compared to the virome in dead pangolin samples (Figure 1a), limited viruses were discovered in fecal samples from the conserved healthy pangolins (Figure 1b). This might be resulted from different sample types. Nevertheless, our study indicated that gastrointestinal viral pathogens, such as the RVs [7, 8], coronaviruses [7, 9], pestiviruses [5, 9], and parvoviruses [10] that were previously reported in pangolins, were not commonly prevalent in captive healthy pangolins in our facility.

Considering above results, future studies on the identification of pangolin viruses should pay attention to two aspects. First, metagenomics has become a fundamental tool to explore viruses in pangolin samples, but the occurrence of possible infections needs to be confirmed by further virological tests (molecular detection, serological survey, and/or virus isolation). Despite our confirmation that pangolin samples contained novel RVD genomes that were divergent from those of existing avian-origin RVD strains (Figure 2), our study still did not show that these newly identified strains could infect pangolins. This was due to both the absence of RVD detected in pangolin fecal samples (Figure S2c,d) and the absence of culturable virus obtained from positive tissue samples. Second, there is a risk of contamination of samples collected from the confiscated wildlife. The pangolin digs up soil to find food, eats termites, and ants with a long sticky tongue, and consumes small stones that aid digestion. It was possible that the pangolin ingested soil contaminated by birds' feces in the wild.

In phylogenetic analyses, according to the NT or amino acid sequences of individual structural proteins or the concatenated genome sequences, two major clades with diverse species of RVs can be clearly defined [13, 26, 30, 31]. RVs can also be divided into the similar two clades and diverse species based on the NT or amino acid sequences of some nonstructural proteins [13, 26, 30]. Our phylogenetic analyses of VP6 gene sequences supported the classification of the two major clades with 11 species, designated as RVA- and RVB-like clades, or clades 1 and 2 (Figure 2a). Clade I consisted of RV species A, C–D, F, and K, and Clade II comprised RV species B, G–J, and L (Figure 2a). It is interesting that RVs, as shown in Figure 2a, identified in our pangolin samples belong to the RVD that has been found only in avian species [22]. Genotypes of RVD were not well classified due to the scarcity of gene sequences. In this study, comprehensive phylogenetic analyses were performed based on VP6 gene sequences of all available RVD strains. According to the results, RVD strains were divided into six exclusive clusters and one distinct strain (Figure 2b–d), which were slightly different from the previous phylogenetic classification [22]. In the previous phylogenetic tree, the Indian RVD sequence was clustered together with Nigerian, German, and Italian RVD sequences into cluster 2.1, and the Brazilian RVD sequences identified before 2013 were classified into two clusters 2.3 and 2.4 [22]. In this study, as new RVD sequences were included, the Indian RVD sequences formed a separate cluster 2.2, the two clusters of Brazilian RVD sequences identified before 2013 were combined together to form a cluster 2.5, and our RVD sequences constituted a new cluster 2.3 (Figure 2b–d).

VP4 and VP7 gene sequences are commonly used to determine the G and P genotypes of RVA isolates [32]. To date, there is limited information on the classification of genotypes of RVD isolates. A previous study showed that RVD sequences fell into two different major clusters based on the VP7 gene sequences [33]. Similar to this finding, our present study found that RVD sequences were divided into two distinct major clusters based on not only the VP7 gene sequences but also the VP1, VP2, VP4, and NSP1–NSP3 gene sequences (Figure 3a,c–g). Interestingly, the VP1, VP2, VP4, and NSP1–NSP3 gene sequences of RVD strains identified in pangolin samples were genetically close to the cluster-representative strain RVD/Chicken/South Korea/D62/2013 (Figure 3a,c,–g and Table 2), while their VP7 gene sequences were genetically close to the Brazilian chicken RVD strains of another cluster (Figure 3b and Table 2). Whether genetic reassortments drive the diversity of RVD strains warrants further investigation.

## 5. Conclusions

In this study, we identified novel RVD strains in pangolin samples. Further sequence alignments and phylogenetic analyses indicated that these RVD strains were divergent from known RVD strains and formed a separate cluster. As a result of these findings, new insights are gained into the RVD's host range and genetic diversity. Furthermore, we monitored potential gastrointestinal viral pathogens in captive healthy pangolins with metagenomic sequencing and molecular detection. Among our pangolin populations, no common gastrointestinal viruses were observed. Despite this, our strategies for monitoring viral pathogens may be applied sustainably to prevent pangolin infectious diseases and even zoonotic transmissions of pangolin viruses.

## Figures and Tables

**Table 1:** Specific primers for amplification of RV genomic segments and detection of RVs A and D.

No.	Name	Sequence (5′−3′)	Fragment (bp)	No.	Name	Sequence (5′−3′)	Fragment (bp)
1	RotaD-NSP1-1-F	AGTGAYTAYGTTTCACAGATGAGATG	700	17	RotaD-VP2-3-F	GTAAATTCAATTCTTTAYCCAGCATTTG	1021
2	RotaD-NSP1-1-R	TGGCTAATCACTGGAATYGATTCC	—	18	RotaD-VP2-3-R	TCGAACGGATTATGTGTYGTAT	—
3	RotaD-NSP1-2-F	GAARCTCRACTAGACCAATGATTACA	921	19	RotaD-VP4-1-F	GGATGTTAGGACCGAAYTCACAAC	616
4	RotaD-NSP1-2-R	TCTATCAACATTATTCCTTCTTCRTCTTC	—	20	RotaD-VP4-1-R	TGCTAAACCRTTTAATTTCCTTGATCTAC	—
5	RotaD-NSP2-F	GCTGCTTCGTAAATGTGGTTGAAT	919	21	RotaD-VP4-2-F	CTAATTACAACCCCAGCRTCAGC	1285
6	RotaD-NSP2-R	CTAATTCACRGTAGACATTTCATCCAG	—	22	RotaD-VP4-2-R	ATCAATTCWGCCATATCATCTGTACC	—
7	RotaD-NSP3-F	CTGAATGGTGCAACCCAGGAAG	1166	23	RotaD-VP6-1-F	CGYTGTCTTCAATTGCGTTGAC	720
8	RotaD-NSP3-R	AGTGTATCCGGTTAAGGGCGAT	—	24	RotaD-VP6-1-R	GCAGCTTGATTTCTARTCATRCT	—
9	RotaD-VP1-1-F	GGGACCTATAAYTTACTCTTACAGAAG	1399	25	RotaD-VP6-2-F	ATACATTTTGCYGCATTTGAYCA	700
10	RotaD-VP1-1-R	TGTTGRGCAATRAAATATGGATATGG	—	26	RotaD-VP6-2-R	CCACATACTCAGCCTAGTTCCAC	—
11	RotaD-VP1-2-F	ATTCCATTAGGAMGRCGTGATGT	685	27	RotaD-VP7-F	ATGCATAACTWTCTWGTGAACAGT	759
12	RotaD-VP1-2-R	TTAGCCATCTCTARTCCTGTMGTAGC	—	28	RotaD-VP7-R	CATATACATTRTARCCTCCAATTTGTAT	—
13	RotaD-VP2-1-F	GTATGAYACTTCAGCAGTTAGGAC	919	29	RotaD—Test-F	TGTGTTGATGATWCTCTAGCRGATTAT	424
14	RotaD-VP2-1-R	CAAATGCTGGRTAAAGAATTGAATTTAC	—	30	RotaD—Test—R	GATGAATCAGCAATRTTRCAACCAAT	—
15	RotaD-VP2-2-F	ATACTGGAGTAATTATGAATATGATTCC	521	31	RotaA—Test-F	TCAACTAATGAGACCWCCAAATATGAC	307
16	RotaD-VP2-2-R	TCGAACGGATTATGTGTYGTAT	—	32	RotaA—Test-R	CATGCTTCTAATGGAAGCCACWGTA	—

Abbreviation: RV, rotavirus.

**Table 2:** Sequence alignments between new and known RVs.

The new RV strain	Gene segment	Obtained length	Complete length	The most closely related known RV strain	Similarity^a^
RVD/Pangolin/China/43/2023	—	1493	—	NC_014515 RVD/Chicken/Germany/05V0049/DEU/2005	93.49%
RVD/Pangolin/China/58/2023	NSP1	816	1872	KM254197 RVD/Chicken/South Korea/D62/2013	93.24%
RVD/Pangolin/China/263/2023	—	1441	—	NC_014515 RVD/Chicken/Germany/05V0049/DEU/2005	93.54%

RVD/Pangolin/China/263/2023	NSP2	810	1026	KM254198 RVD/Chicken/South Korea/D62/2013	95.73%

RVD/Pangolin/China/58/2023	NSP3	1079	1270	KM254199 RVD/Chicken/South Korea/D62/2013	95.84%
RVD/Pangolin/China/263/2023	—	1103	1242	—	96.05%

RVD/Pangolin/China/43/2023	—	1897	—	KM254191 RVD/Chicken/South Korea/D62/2013	90.78%
RVD/Pangolin/China/58/2023	VP1	625	3274	—	91.17%
RVD/Pangolin/China/263/2023	—	1899	—	—	91.10%

RVD/Pangolin/China/43/2023	—	1810	—	KM254192 RVD/Chicken/South Korea/D62/2013	97.29%
RVD/Pangolin/China/58/2023	VP2	835	2801	—	97.59%
RVD/Pangolin/China/263/2023	—	1830	—	—	96.39%

RVD/Pangolin/China/43/2023	—	1685	—	KM254194 RVD/Chicken/South Korea/D62/2013	95.55%
RVD/Pangolin/China/58/2023	VP4	540	2366	—	95.37%
RVD/Pangolin/China/263/2023	—	1722	—	—	95.59%

RVD/Pangolin/China/43/2023	—	1202	—	JN034683 RVD/Chicken/Bangladesh/BGD/BS-7/2010	90.18%
RVD/Pangolin/China/58/2023	VP6	760	1353	—	90.17%
RVD/Pangolin/China/263/2023	—	1173	—	—	91.21%

RVD/Pangolin/China/43/2023	VP7	655	1052	KJ101578 RVD/Chicken/Brazil/BRA/98/2010	89.01%
RVD/Pangolin/China/263/2023	—	739	—	KJ101575 RVD/Chicken/Brazil/BRA/85/2010	89.97%

Abbreviations: RV, rotavirus; RVD, rotavirus species D.

^a^Similarity: The sequence similarity of a gene segment between the new RV strain found in this study and the most homologous known RV strain to it.

## Data Availability

The datasets described in this study can be found in online repositories. The data of virome studies on pangolin samples were deposited in the NCBI database under BioProject ID: PRJNA1084880. The gene segments sequences of novel RVD strains in pangolin samples were submitted to GenBank under accession numbers PP319611-PP319630.

## Supporting Information

Supporting Information 1 Figure S1: Molecular detection of rotaviruses in pangolin samples. Agarose gel electrophoresis analysis of the amplicon products resulting from RT-PCR on dead pangolin samples (a,b) or healthy pangolin fecal samples (c, d) using the primers targeting the VP7 gene of rotavirus D (a and c) or the VP6 gene of rotavirus A (b and d). “–”: negative controls; “+”: positive controls; “ZXY” or “MXY”: Z, MM, X, and Y represent the Chinese pangolin, Malayan pangolin, animal number, and sample number, respectively. Two or three RNA samples from pangolin feces were mixed for the detection of rotavirus D or A.

Supporting Information 2 Figure S2: Gene segments amplification of rotaviruses in pangolin samples. Agarose gel electrophoresis exhibiting the amplicon products of individual gene segments of rotaviruses identified from dead pangolin samples #263 (a), #43 (b), and #58 (c).

Supporting Information 3 Figure S3: The mean pairwise patristic distances between sequences within or among individual RVD clusters. The RVD VP6 gene sequences were aligned by ClustalW in MEGA-X. The pairwise patristic distances between sequences were exported following the phylogenetic analysis. (a) The mean pairwise patristic distances between sequences within individual clusters. (b) The average pairwise patristic distances between sequences among clusters and the distinct strain.
