# Supplementary figures and images for: Identification and Genetic Analysis of Species D Rotaviruses in Pangolin Samples
Source: Transbound Emerg Dis. 2024 Nov 26;2024:1773821. doi: 10.1155/tbed/1773821 (PMC12016870; doi:10.1155/tbed/1773821)

Figure S1

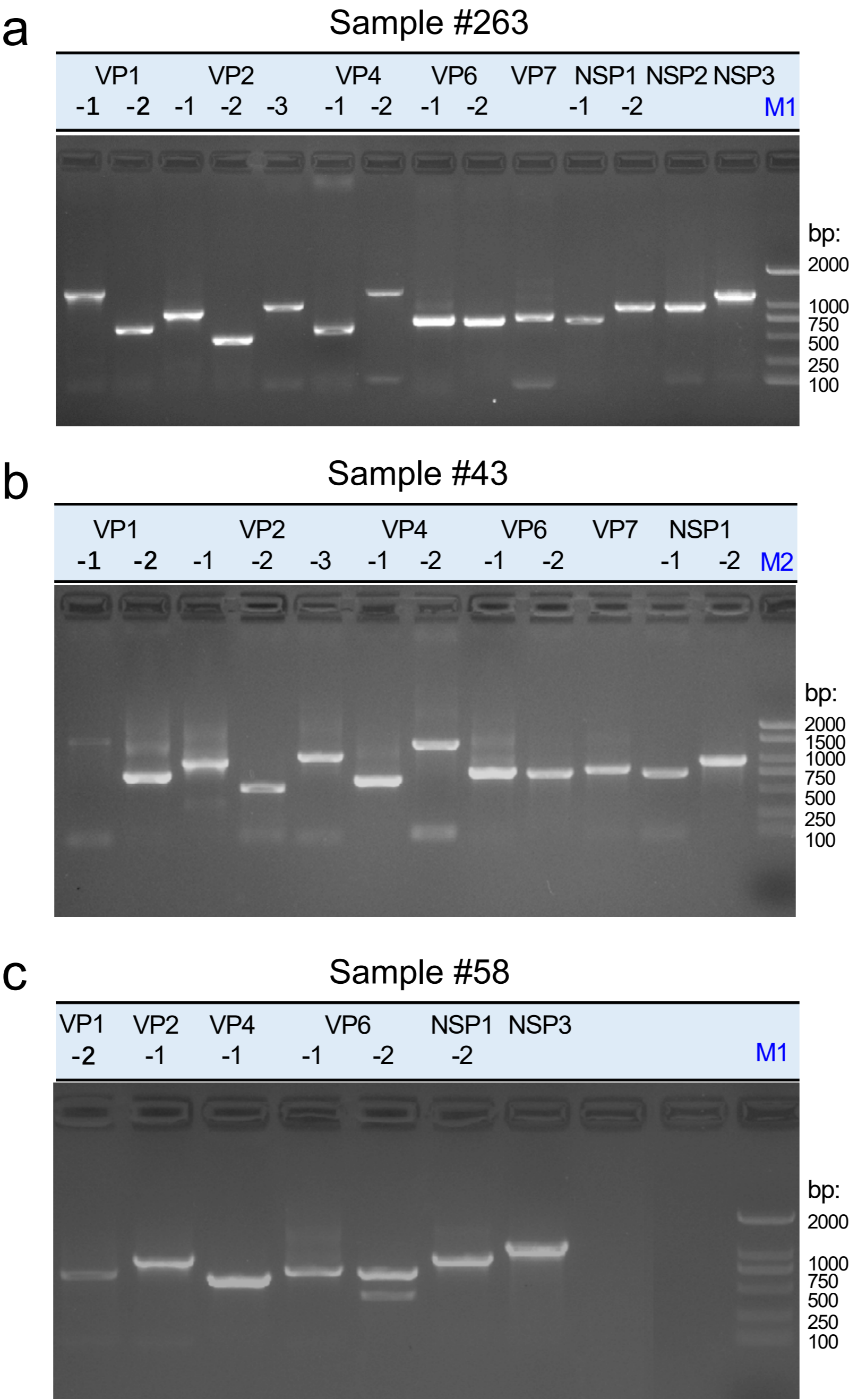

Supplement: Supporting Information 1 — Figure S1: Molecular detection of rotaviruses in pangolin samples. Agarose gel electrophoresis analysis of the amplicon products resulting from RT-PCR on dead pangolin samples (a,b) or healthy pangolin fecal samples (c, d) using the primers targeting the VP7 gene of rotavirus D (a and c) or the VP6 gene of rotavirus A (b and d). “–”: negative controls; “+”: positive controls; “ZXY” or “MXY”: Z, MM, X, and Y represent the Chinese pangolin, Malayan pangolin, animal number, and sample number, respectively. Two or three RNA samples from pangolin feces were mixed for the detection of rotavirus D or A. [file 1773821.f1.pdf]

Figure S2

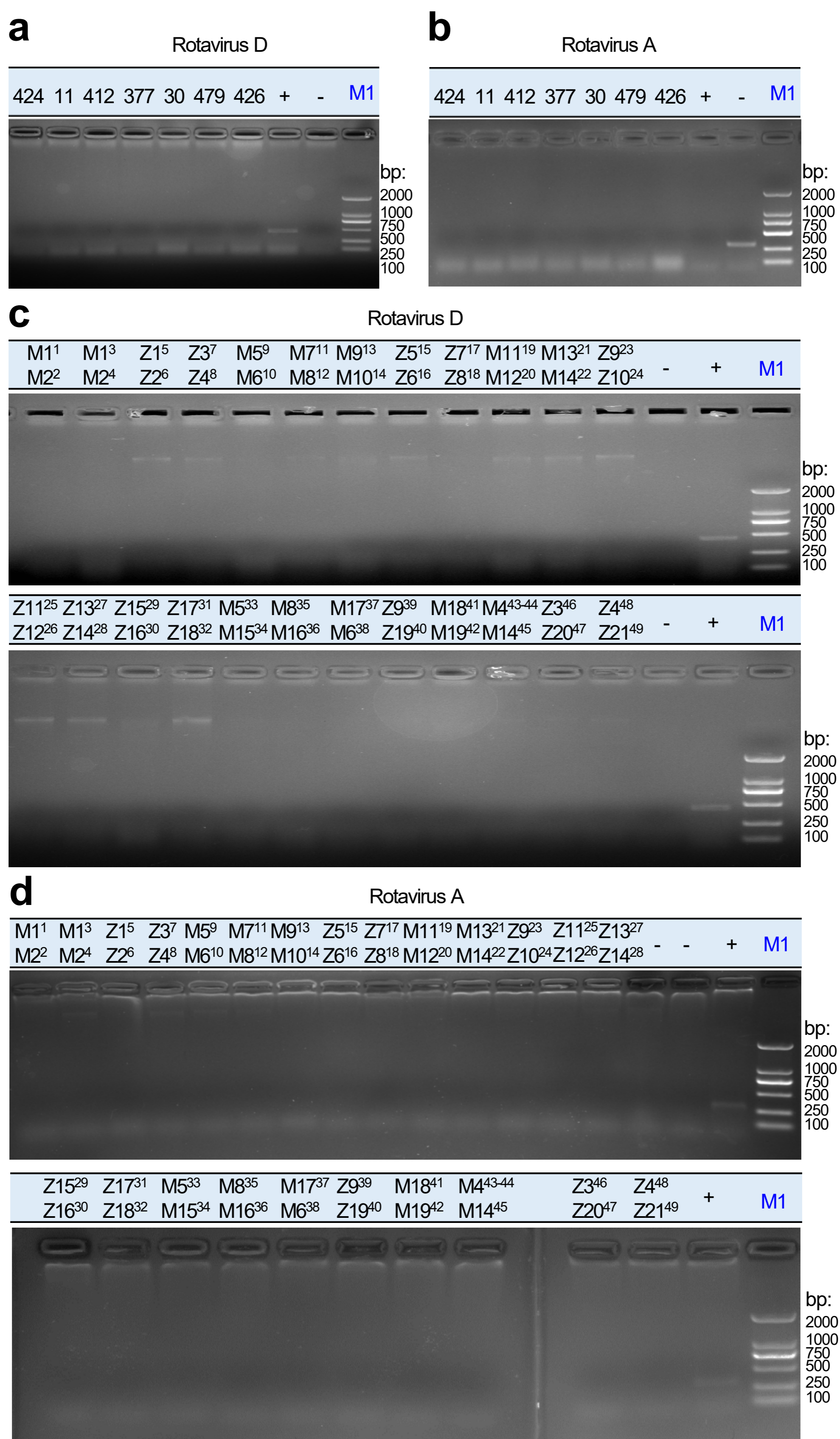

Supplement: Supporting Information 2 — Figure S2: Gene segments amplification of rotaviruses in pangolin samples. Agarose gel electrophoresis exhibiting the amplicon products of individual gene segments of rotaviruses identified from dead pangolin samples #263 (a), #43 (b), and #58 (c). [file 1773821.f2.pdf]
