# Supplementary material for: Identification and Genetic Analysis of Species D Rotaviruses in Pangolin Samples
Source: Transbound Emerg Dis. 2024 Nov 26;2024:1773821. doi: 10.1155/tbed/1773821 (PMC12016870; doi:10.1155/tbed/1773821)
Supplement: Supporting Information 3 — Figure S3: The mean pairwise patristic distances between sequences within or among individual RVD clusters. The RVD VP6 gene sequences were aligned by ClustalW in MEGA-X. The pairwise patristic distances between sequences were exported following the phylogenetic analysis. (a) The mean pairwise patristic distances between sequences within individual clusters. (b) The average pairwise patristic distances between sequences among clusters and the distinct strain. [file 1773821.f3.pdf]

Figure S3

a

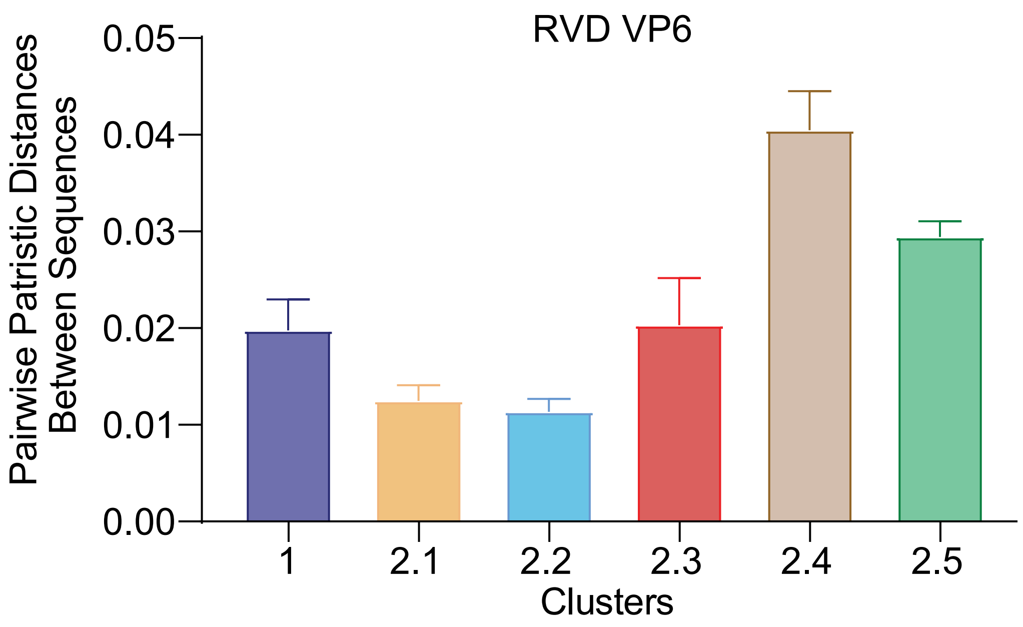

b

The average pairwise patristic distances between sequences among clusters and the distinct strain

	Cluster 1	Cluster 2.1	Cluster 2.2	Cluster 2.3	Cluster 2.4	Cluster 2.5	Distinct strain
Cluster 1							
Cluster 2.1	0.138						
Cluster 2.2	0.165	0.133					
Cluster 2.3	0.167	0.135	0.146				
Cluster 2.4	0.159	0.128	0.139	0.120			
Cluster 2.5	0.168	0.136	0.148	0.129	0.114		
Distinct strain	0.224	0.237	0.264	0.265	0.258	0.267	
